# Alterations to Cerebral Perfusion, Metabolite Profiles, and Neuronal Morphology in the Hippocampus and Cortex of Male and Female Mice during Chronic Exposure to a High-Salt Diet

**DOI:** 10.3390/ijms24010300

**Published:** 2022-12-24

**Authors:** Anja Meissner, Alba M. Garcia-Serrano, Lotte Vanherle, Zeinab Rafiee, Nicholas Don-Doncow, Cecilia Skoug, Sara Larsson, Michael Gottschalk, Martin Magnusson, João M. N. Duarte

**Affiliations:** 1 Department of Experimental Medical Science, Faculty of Medicine, Lund University, 22184 Lund, Sweden; 2 Wallenberg Centre for Molecular Medicine, Lund University, 22184 Lund, Sweden; 3 Department of Physiology, Institute of Theoretical Medicine, Medical Faculty, University of Augsburg, 86159 Augsburg, Germany; 4 Lund University Bioimaging Center, Lund University, 22184 Lund, Sweden; 5 Department of Clinical Sciences, Lund University, 20502 Malmö, Sweden; 6 Department of Cardiology, Skåne University Hospital, 20502 Malmö, Sweden; 7 Hypertension in Africa Research Team (HART), North-West University, Potchefstroom 2520, South Africa

**Keywords:** neurodegeneration, metabolism, hypertension, sodium, CBF, MRS

## Abstract

Excess dietary salt reduces resting cerebral blood flow (CBF) and vascular reactivity, which can limit the fueling of neuronal metabolism. It is hitherto unknown whether metabolic derangements induced by high-salt-diet (HSD) exposure during adulthood are reversed by reducing salt intake. In this study, male and female mice were fed an HSD from 9 to 16 months of age, followed by a normal-salt diet (ND) thereafter until 23 months of age. Controls were continuously fed either ND or HSD. CBF and metabolite profiles were determined longitudinally by arterial spin labeling magnetic resonance imaging and magnetic resonance spectroscopy, respectively. HSD reduced cortical and hippocampal CBF, which recovered after dietary salt normalization, and affected hippocampal but not cortical metabolite profiles. Compared to ND, HSD increased hippocampal glutamine and phosphocreatine levels and decreased creatine and choline levels. Dietary reversal only allowed recovery of glutamine levels. Histology analyses revealed that HSD reduced the dendritic arborization and spine density of cortical and hippocampal neurons, which were not recovered after dietary salt normalization. We conclude that sustained HSD exposure throughout adulthood causes permanent structural and metabolic alterations to the mouse brain that are not fully normalized by lowering dietary salt during aging.

## 1. Introduction

Sodium is the principal cation in extracellular body fluids and is necessary for the maintenance of plasma volume, acid–base balance, neurotransmission, and other normal cell functions. However, excessive sodium intake, mainly from dietary salt, is known to be associated with high blood pressure (hypertension) and increased cardiovascular risk [1], including cerebrovascular derangements [2,3] that contribute to cognitive impairment [4]. Reduction of dietary sodium intake lowers blood pressure in both hypertensive and non-hypertensive individuals [5]. 

Evidence has emerged that both high and low blood pressure play a role in the development and progression of cognitive impairment and dementia, depending on age [6], and many studies have reported an association between hypertension in midlife and development of dementia and Alzheimer’s disease later in life [6,7]. Others have demonstrated that both orthostatic hypotension, a manifestation of autonomic failure (insufficient hemodynamic adaptation to postural changes), and symptoms of orthostatic intolerance may predict cognitive decline [8,9,10]. Furthermore, recent meta-analyses indicate that blood pressure lowering with antihypertensive agents is associated with a lower risk of incident dementia or cognitive impairment [11,12]. In contrast, a decline in blood pressure between middle and old age appeared to be a risk factor for developing dementia [9]. Population studies are often impacted by low power due to the low incidence of dementia, and some degree of controversy has emerged regarding the association between salt intake and cardiovascular disease, as well as cognition [13,14]. In turn, rodent studies have found impairments in cognition following consumption of high-salt (typically 7–8%) diets for 1–3 months [14]. Interestingly, a high-salt diet (HSD) appears to induce hypertension in rats [15,16] but not in mice [17,18]. Nevertheless, in mice, HSD affects the cerebral vasculature and impairs memory in the absence of blood pressure changes [17,19,20], suggesting blood-pressure-independent mechanisms of brain dysfunction. Furthermore, Faraco et al. have suggested that neurovascular defects caused by HSD exposure for 3 months are reversible by returning dietary salt intake to normal levels in young adult mice [19]. Such short-term salt exposure does not mimic sustained high-salt intake in humans, and age is likely the major determinant of the divergent blood pressure responses to increasing salt intake, with general salt sensitivity being higher in older than in younger subjects [21]. Moreover, neurovascular impairments are likely to result in poor fueling of brain metabolism, which is critical for neuronal function ([22] and references therein). It remains unknown whether metabolic and cerebrovascular derangements developed during long-term HSD exposure in adulthood can be reversed by reducing dietary salt content during aging. 

Metabolite profiling by magnetic resonance spectroscopy (MRS) in vivo provides putative biomarkers of aging and neurodegenerative processes [23]. MRS studies during diet interventions showed that metabolism in the central nervous system (CNS) is impacted by dietary contents of fat and sugar [24,25,26], but the impact of HSD on cerebral metabolite profiles in vivo is hitherto unknown. Thus, the present study aimed at investigating the impact of long-term HSD exposure on cerebral perfusion and metabolic profiles of the cortex and hippocampus. We further tested the reversibility of metabolite alterations by normalization of dietary salt intake and whether metabolite changes were accompanied by neurodegeneration.

## 2. Results

**Figure 1:**
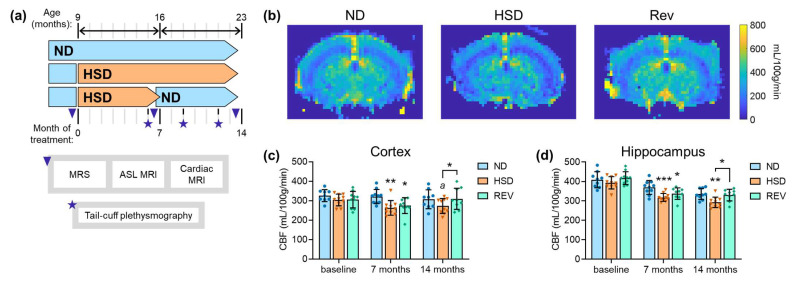
Exposure to high-salt diet (HSD) during aging leads to lower cortical and hippocampal perfusion in mice, which recover upon normalization of dietary salt intake. (**a**) Study design for diet intervention over 14 months. (**b**) Representative cerebral blood flow (CBF) maps measured by ASL-MRI from mice fed with normal diet (ND) or HSD for 14 months, or HSD followed by diet normalization (Rev). (**c**,**d**) Mean cortical (**c**) and hippocampal (**d**) CBF at baseline, before diet normalization (i.e., 7 months) and at the end of the study (i.e., 14 months), as measured by ASL-MRI (n = 9–10). Data are means ± SDs. Symbols overlaid on bars represent independent measurements from each mouse. * *p* < 0.05, ** *p* < 0.01, *** *p* < 0.001, *^a^ p* = 0.065 compared to ND or as indicated using Fisher LSD tests after significant effects of diet were determined by two-way ANOVA.

Mice were fed a normal-salt diet (ND) containing 0.6% NaCl or an HSD consisting of a chow diet with additional 8% (*w*/*w*) NaCl from 9 to 23 months of age. In parallel, a reversed diet group (Rev) received HSD for 7 months, followed by ND until 23 months of age (Figure 1A). In both male and female mice, HSD-feeding reduced weight gain, which recovered upon diet reversal (Table 1). In turn, average food intake was increased for mice subjected to HSD relative to the low-salt group, especially in males (at least a 50% increase as compared to only a 20–25% increase in females; Table 1). HSD consumption was accompanied by a concomitant 2–3-fold increase in water consumption (Table 1). These observations are in line with previous reports (e.g., [20]). 

### 2.1. Cerebral Blood Flow

First, we tested whether CBF was impacted by long-term HSD exposure (Figure 1B). HSD feeding for 7 and 14 months resulted in reduced cortical perfusion, which was reversed after dietary salt normalization (Figure 1C). Similar effects of HSD exposure were observed in the hippocampus and, in addition, hippocampal CBF was significantly reduced with age (Figure 1D). These CBF alterations were not paralleled by alterations in blood pressure, heart rate, or cardiac left ventricular ejection fraction (Table 2), suggesting that cerebral perfusion alterations are independent of systemic hemodynamic profiles in response to HSD in aging mice. 

### 2.2. Brain Metabolite Concentrations

**Figure 2:**
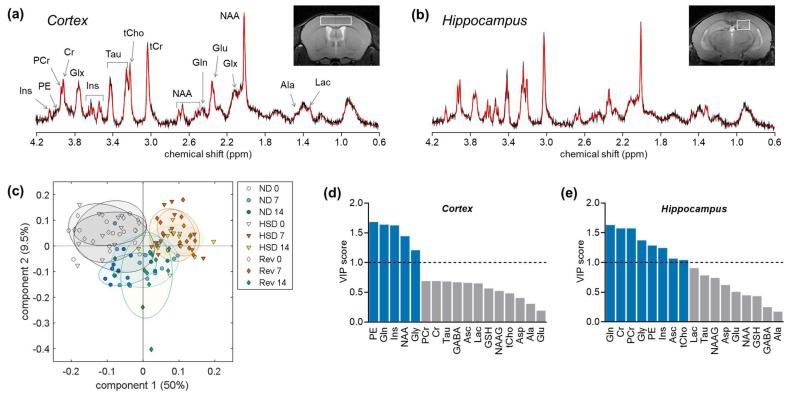
Metabolic profiles of cortex and hippocampus in response to HSD and aging. Representative MRS spectra in the cortex (**a**) and hippocampus (**b**) acquired with STEAM at 9.4 T overlaid by the respective LCModel fitting results (red lines). The VOI locations in the cortex and dorsal hippocampus are represented in the inset anatomical images. Most prominent peaks are labelled: Ala, alanine; Lac, lactate; GABA, γ-aminobutyrate; NAA, *N*-acetylaspartate; Glx, glutamate (Glu) + glutamine (Gln); tCr, total creatine = creatine (Cr) + phosphocreatine (PCr); tCho, total choline = phosphorylcholine + glycerophosphorylcholine; Tau, taurine; Ins, *myo*-inositol; PE, phosphorylethanolamine. The resulting neurochemical profile was analyzed with a PLS regression to the presence of high salt concentrations in the diet. (**c**) Component space after PLS regression showing individual mice (symbols) and group SDs (ellipsoids) for normal (ND), high-salt (HSD), and reversed (Rev) diet groups at 0, 7, and 14 months of treatment. (**d**,**e**) VIP scores calculated from the resulting PLS model for metabolites in the cortex and hippocampus.

^1^H spectra for the cortex and hippocampus were analyzed using LCModel to determine metabolite profiles (Figure 2A,B). The mice in this study showed the known changes in creatine concentration during aging [27], but total creatine was not significantly altered by diet in either the cortex (ANOVA: age F(2,104) = 3.44, *p* = 0.036; diet F(2,104) = 1.03, *p* = 0.360; interaction F(4,104) = 0.455, *p* = 0.768) or the hippocampus (ANOVA: age F(2,106) = 16.90, *p* < 0.001; diet F(2,106) = 0.173, *p* = 0.842; interaction F(4,106) = 0.491, *p* = 0.742). Thereafter, in order to minimize variance across measurements, metabolite concentrations were analyzed as ratios to total creatine (tCr). Then, z-scores for metabolite concentrations from all groups and ages were analyzed altogether with a two-component partial least-squares (PLS) regression for the presence of high amounts of dietary salt. PLS regression revealed an effect of age and allowed good separation of HSD-fed mice from groups fed a low-salt diet (Figure 2C), with PC1 and PC2 accounting for 50% and 9.5% of the variance in metabolite levels. The variable importance in projections (VIPs) calculated from the PLS model coefficients (Figure 2D,E) indicated that HSD and/or aging were associated with modifications (VIP > 1) to glutamine, glycine, phosphorylethanolamine, and *myo*-inositol levels in both the hippocampus and the cortex. HSD and/or aging were also associated with modifications to creatine, phosphocreatine, ascorbate, total choline, and *N*-acetylaspartate levels in the hippocampus. The following analyses focused on metabolite concentrations with VIPs > 1.

**Figure 3:**
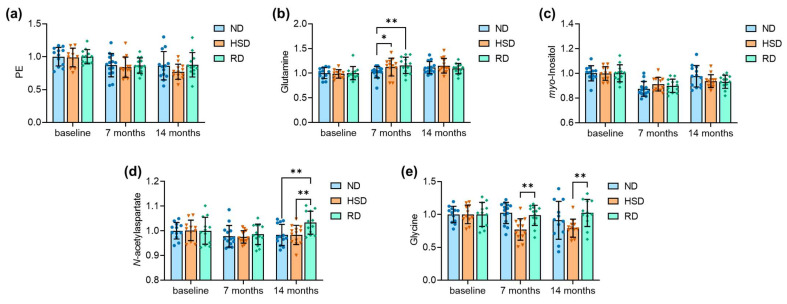
Cortical VIP metabolite concentrations for PE (**a**), glutamine (**b**), *myo*-inositol (**c**), N-acetylaspartate (**d**) and glycine (**e**) representing the main age- and/or diet-induced alterations to metabolite profiles. Data are normalized to the mean baseline concentration within each experimental group. Bars are means ± SDs. Symbols overlaid on bars represent independent measurements from each mouse for normal (ND), high-salt (HSD), and reversed (Rev) diet groups. * *p* < 0.05, ** *p* < 0.01, compared to ND or as indicated using Fisher LSD tests after significant effects of diet were determined by two-way ANOVA.

In the cortex, phosphorylethanolamine concentrations were prominently reduced with age but not affected by HSD (Figure 3A). Cortical glutamine levels showed an increase with age and tended to increase faster in mice exposed to HSD (Figure 3B). The aging process was also associated with changes in *myo*-inositol concentrations, with no impact of HSD (Figure 3C). Concentrations of *N*-acetylaspartate were reduced with age and particularly increased after diet reversal (Figure 3D). Unexpectedly, glycine levels in the cortex were reduced after 7 months of HSD in only one of the experimental groups (Figure 3E), a change that persisted at 14 months of treatment, suggesting that HSD may not be the only contributor to glycine concentration changes in the cortex. We were, however, unable to identify a cause of glycine concentration changes other than HSD feeding.

**Figure 4:**
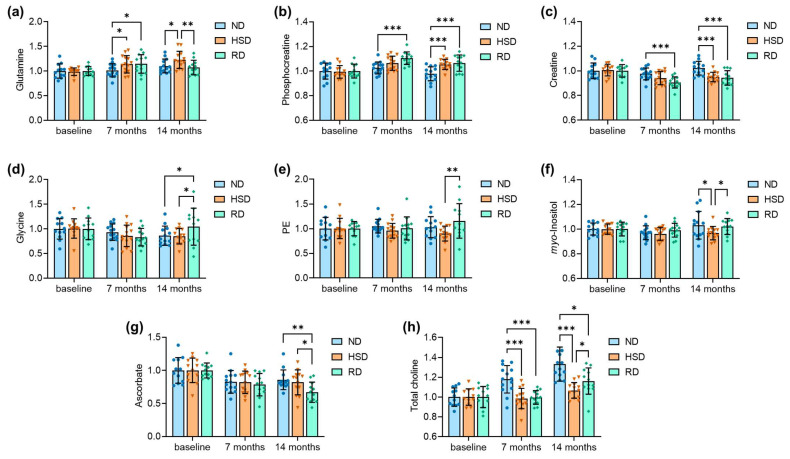
Hippocampal VIP metabolite concentrations for glutamine (**a**), phosphocreatine (**b**), creatine (**c**), glycine (**d**), PE (**e**), *myo*-inositol (**f**), ascorbate (**g**) and total choline (**h**) representing major age- and/or diet-induced alterations to the metabolite profiles. Data are normalized to the mean baseline concentration within each experimental group. Bars are means ± SDs. Symbols overlaid on bars represent independent measurements from each mouse for normal (ND), high-salt (HSD), and reversed (Rev) diet groups. * *p* < 0.05, ** *p* < 0.01, *** *p* < 0.001 compared to ND or as indicated using Fisher LSD tests after significant effects of diet were determined by two-way ANOVA.

In the hippocampus, HSD and age caused an increase in levels of glutamine, which was normalized after diet reversal (Figure 4A), although ANOVA mainly depicted aging effects. HSD also increased phosphocreatine levels (Figure 4B), with a concomitant reduction in creatine levels (Figure 4C), which did not normalize after diet reversal. Hippocampal glycine levels were mainly affected by aging but increased upon diet reversal (Figure 4D). Although statistically significant differences were not observed for levels of phosphorylethanolamine in the hippocampus, there was a general tendency to reduction with HSD and recovery after diet reversal (Figure 4E). Similarly, *myo*-inositol levels did not reach significance in ANOVA; a tendency (significantly different in post-testing after ANOVA) to reduction by HSD at 14 months relative to the ND and reversed diet groups was observed (Figure 4F). Age reduced levels of ascorbate in the hippocampus, which were particularly low after diet reversal (Figure 4G). Total choline increased with age in the ND group, but not upon HSD feeding, and there was minimal recovery upon diet reversal (Figure 4H).

Altogether, metabolite profiles in the cortex and the hippocampus changed with age, were impacted by HSD, but did not recover upon diet reversal to normal salt concentrations. Interestingly, reversing HSD was associated with specific changes, including increased cortical *N*-acetylaspartate and increased hippocampal glycine concentrations.

### 2.3. Neuronal Morphology

**Figure 5:**
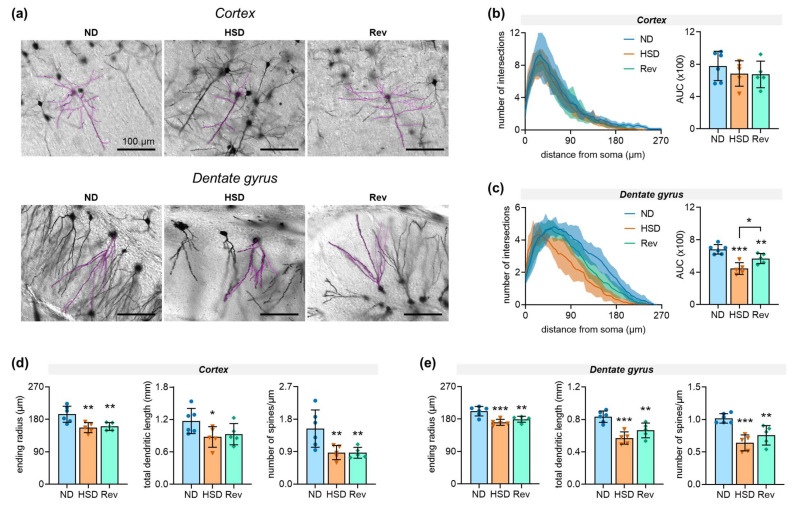
Neuronal morphology after 14 months feeding with normal diet (ND), high-salt diet (HSD), or HSD followed by diet reversal (Rev). (**a**) Representative micrographs of Golgi–Cox-stained pyramidal neurons in the cortex and granule cells in the dentate gyrus of the hippocampus. Dendritic morphologies in typical Sholl analyses are highlighted by the superimposed magenta traces. (**b**,**c**) Sholl analysis showing number of dendrite intersections (i.e., dendritic branching) *vs*. distance from neuronal soma. (**d**,**e**) Maximal dendritic length (i.e., ending radius), total dendritic length, and dendritic spine density (number of spines on third-order branch) for neurons in the cortex and dentate gyrus. Data are means ± SDs of n = 5–6. Symbols overlaid on bars represent independent measurements from each mouse. * *p* < 0.05, ** *p* < 0.01, *** *p* < 0.001 compared to ND by Fisher LSD tests after significant results were determined by one-way ANOVA. Bars over micrographs represent 100 µm.

In order to identify whether metabolite alterations were accompanied by structural alterations to neurons, we analyzed the morphologies of pyramidal cells in the cortex and granule cells in the dentate gyrus of the hippocampus after Golgi–Cox staining (Figure 5A). In the cortex, pyramidal cells showed similar branching patterns across the experimental groups (Figure 5B). In turn, granule cells from the hippocampi of HSD-fed mice showed lower numbers of intersections than those in ND-fed mice (Figure 5C). In both cortical and hippocampal neurons, HSD feeding for 14 months caused a reduction in maximal dendritic length, total dendritic length, as well as number of dendritic spines (Figure 5D,E). Overall, normalization of dietary salt intake was unable to promote complete recovery of neuronal morphology alterations, despite some improvement in the morphologies of granule cells of the dentate gyrus.

## 3. Discussion

This study demonstrates that after sustained HSD feeding throughout adulthood, a protocol that mimics human excessive salt intake, long-term neuronal damage is not recoverable. HSD further caused a reduction in resting CBF in the cortex, and most strikingly in the hippocampus, which was recovered after diet salt normalization. On the contrary, lowering of salt intake did not recover metabolite profiles measured by MRS to the pattern observed in age-matched ND-fed mice.

Metabolite profile changes in the cortex were mainly related to aging and were in general agreement with previous MRS studies in aged mice (see [27] and references therein). An exception was cortical glutamine, levels of which increased with aging, but the levels increased at an earlier age in HSD-fed mice relative to those under ND. Cerebral glutamine is mainly synthetized in astrocytes, via glutamine synthetase, and increased cortical levels of glutamine are often acknowledged to represent the gliosis process during aging (discussed in [23]). However, *myo*-inositol concentration changes did not parallel those of glutamine. The concentration of *myo*-inositol in the cortex has been taken as a robust astrogliosis marker in Alzheimer’s disease, although it does not correlate with protein markers of gliosis in brain areas other than the cortex (discussed in [23]). In the present study, cortical *myo*-inositol levels were reduced from 9 to 16 months of age and then increased at 23 months, reproducing previous findings [27]. Altogether, these observations and the absence of an HSD-induced *myo*-inositol increase suggests that high dietary salt intake did not trigger astrogliosis in our model. 

HSD feeding affected the metabolite profile of the hippocampus. Most strikingly, HSD increased glutamine concentrations, which recovered after normalizing dietary salt consumption. Glutamine accumulates in the brain as a mechanism for ammonia detoxification [28], as well as upon metabolic remodeling after re-oxygenation in a transient ischemia model [29]. It is plausible that injury to the kidneys and liver during long-term HSD feeding (over 7 months in our study) leads to ammonia excretion deficits and consequently to brain glutamine accumulation. However, kidney injury is likely to occur with the development of hypertension, which has not been reported in mice subjected to HSD (see [17,18] and Figure 1). A recent study on mice showed that four-week high-sodium intake stimulated arginase activity in the liver and skeletal muscle and increased urea concentrations in the blood [30]. These findings suggest a preserved urea cycle for blood ammonia removal within the first month of HSD exposure, although it is unknown whether this was impaired in our study. Increased cerebral glutamine levels, as measured in our study, were found to accompany electrophysiological deficits, brain atrophy, and behavioral impairments in mouse models of Huntington’s disease [31] and schizophrenia [32].

Hippocampal levels of the putative gliosis marker *myo*-inositol tended to show changes that were opposite to those of glutamine, namely, *myo*-inositol was reduced by HSD after 14 months, but not after diet normalization. It is therefore unlikely that HSD induces hippocampal gliosis. In line with this, a relatively shorter HSD feeding paradigm did not induce gliosis and neuroinflammation in mice [33,34]. On the contrary, HSD-associated astrocyte reactivity was reported to occur in the brains of rats [35] that developed hypertension in response to excessive dietary salt intake [15,16]. Nonetheless, a metabolic dysfunction in astrocytes is likely to result in poor metabolic support to neurons, leading to synaptic degeneration, especially glutamatergic synapses, even in the absence of astrogliosis [22]. Studies on rodents subjected to short-term HSD feeding (2–3 months) found loss of dendritic spines in the absence of neuronal death [16] and reduced levels of synaptic proteins [18,20]. This is in line with our observation that HSD for 14 months leads to deteriorated dendritic arborization in cortical and hippocampal neurons, as well as reduced spine density. 

There was an increase in phosphocreatine accompanied by reduced creatine levels, suggesting increased energy availability within the hippocampus, which was not recoverable by diet normalization. However, the brain cells responsible for this higher energy availability remain to be identified. In line with increased cellular energy production, Ge et al. reported increased activity of citrate synthase, the rate-limiting enzyme of the tricarboxylic acid (TCA) cycle, and reduced hexokinase activity in the hippocampi of mice fed an HSD [18]. A recent metabolomics study also reported increased levels of citrate and other TCA cycle intermediates in the brains of aged mice exposed to HSD for 3 months (e.g., [20]). (However, care should be taken when interpreting this dataset reported by Yuan et al., since the sample collection method is not described and there might have been substantial post-mortem metabolic degradation.) 

In line with previous reports [27], total choline levels in the hippocampus increased with age in the ND group. Interestingly, this age-dependent choline increase was blunted in HSD-fed mice. The MRS-observed choline peaks represent mobile choline-containing lipids and have been proposed to depict cell membrane dynamics upon neuroinflammation [36]. The absence of choline increase in HSD-fed mice is thus in line with the above-discussed absence of neuroinflammation. Moreover, choline may also represent the mobilization of phosphatidylcholine from the cell membranes of other cells [37]. Low mobilization of choline-containing lipids from neuronal plasma membranes may be related to dendrite retraction and loss of dendritic spines. Accordingly, hippocampal choline levels trended to recover after diet reversal, paralleling a trend of recovery of dendritic arborization and spine density. 

In our study, weight gain was halted in mice fed the HSD, which was likely due to the induction of catabolic pathways and loss of muscle [30] and (mainly) fat mass [38]. In particular, Cui et al. reported that fat mass, heat production, oxygen consumption rates, and the respiratory exchange ratios in mice fed an HSD were significantly decreased compared with mice fed an ND [38]. Moreover, high salt intake prevented obesity development during high-fat-diet exposure in both male and female mice, although it did not prevent the emergence of fat-induced metabolic dysfunction typified by glucose intolerance and loss of insulin sensitivity [39]. While obesity is almost always one of the strongest metabolic syndrome factors associated with cardiovascular disease [40], the impacts of reduced fat depots and concomitant peripheral metabolism adaptations on the brain remain to be determined. Interestingly, there is evidence that HSD can induce gut inflammation in mice [41], which in turn impacts digestion and nutrient absorption. Moreover, HSD is proposed to disrupt gut microbial activity, leading to increased susceptibility to development of metabolic and inflammatory disorders [42]. 

In sum, previous evidence from rodent studies indicates that high salt intake impairs aspects of cognitive function even in the absence of hypertension (reviewed in [14]). Accordingly, we did not find substantial blood pressure changes in HSD-fed mice. The present results lead us to conclude that chronic HSD consumption in adulthood induces a reversible cerebrovascular perfusion deficit and causes permanent, non-recoverable neuronal injury. These events are accompanied by metabolite profile alterations, in the cortex and hippocampus, most prominently in the latter. 

## 4. Materials and Methods

### 4.1. Animals

Experiments were performed according to EU Directive 2010/63/EU, approved by the Malmö/Lund Committee for Animal Experiment Ethics (#7143/2018), and are reported following the ARRIVE 2.0 guidelines (Animal Research: Reporting In Vivo Experiments, NC3Rs initiative, London, UK). Sample size was estimated from previous MRS studies [25,26]. 

Male and female C57BL/6JRj mice (5–6 months old) were purchased from Janvier Labs (Saint-Berthevin, Le Geneste-Saint-Isle, France) and housed on a 12 h light–dark cycle with lights on at 07:00, room temperature of 21–23 °C and humidity at 55–60% in ventilated cages (522.6 cm² floor space, 12.7 cm height; Innovive, San Diego, CA USA) enriched with corn-cob bedding, nesting paper sheets, climbing ladders, cardboard cylinders, and wood blocks. 

Cages were randomly assigned to one of the following diet interventions for a total duration of 14 months: normal-salt diet (ND) RM3-P (Scanbur, Karlslunde, Denmark) containing 0.6% NaCl (n = 12 males + 15 females), HSD consisting of chow diet with additional 8% (*w*/*w*) NaCl (Scanbur) for 14 months (n = 13 males + 13 females), or a reversed diet (Rev) that consisted of HSD feeding for 7 months followed by ND feeding for 7 months (n = 9 males + 10 females). Food and water were provided ad libitum. Diet interventions started at 9 months of age, and diet normalization was realized at 16 months of age (Figure 1A). Food and water intake were recorded for a month after starting the diet intervention (i.e., at 9 months of age) and at the time of diet reversal (i.e., at 16 months of age). 

### 4.2. Magnetic Resonance Imaging (MRI) and Spectroscopy (MRS)

Arterial spin labeling (ASL) MRI, cardiac MRI, and MRS were performed in 3 different sessions with 3–4 days of interval at baseline, as well as at 7 and 14 months after diet intervention started. Experiments were performed on a 9.4 T Bruker BioSpec AV III (Bruker, Ettlingen, Germany) with an effective bore size of 86 mm and gradient strength of 670 mT/m, using ParaVision 6.0.1 (RRID:SCR_001964), using a quadrature volume resonator coil for transmission and a 20 mm linear surface loop coil for reception for ASL and cardiac MRI, or a ^1^H quadrature transmit-receive cryoprobe for MRS.

Mice were anesthetized with isoflurane (Vetflurane, Virbac, Carros, France) in a 1:1 (*v*/*v*) O_2_:N_2_O gas mixture. For ASL MRI and MRS, mice were positioned on an MRI-compatible bed with tooth and ear bars for stereotaxic fixation. For cardiac MRI, mice were placed in supine position and electrodes were placed for electrocardiography (ECG). ECG, breathing rate, and body temperature were recorded with the SA Instruments (Stony Brook, NY, USA) monitoring system. Anesthesia was delivered through a mask at a variable rate of 1.8–2.5% isoflurane to maintain respiration at 70–100 breaths per minute. Body temperature was kept between 36 and 37 °C by means of warm water circulation. 

T_2_-weighted images were acquired for anatomical reference using the Rapid Imaging with Refocused Echoes (RARE) sequence with repetition time TR = 3.5 s, echo time TE = 33 ms, 32 slices, thickness of 0.5 mm, 320 × 320 voxels, and FOV = 14 × 14 mm^2^. After MAPSHIM (Bruker) and iterative linear shimming, proton MRS was acquired using STimulated Echo Acquisition Mode (STEAM) with TR = 4 s, TE = 3 ms, mixing time TM = 20 ms, and a spectral width of 4401.41 Hz. Volumes of interest (VOIs) were placed in the dorsal hippocampus (1.8 mm × 1.2 mm × 1.5 mm) and cortex (4 mm × 0.8 mm × 1.5 mm). Water-suppressed spectra were acquired in 20 and 12 blocks of 16 scans for the hippocampus and cortex, respectively. Unsuppressed water spectra were acquired from the same VOIs in one block of 16 averages. After block alignment and summation, metabolite concentrations were determined with LCModel v.6.3-1A (Stephen Provencher Inc., Oakville, Ontario-Canada; RRID:SCR_014455), including a macromolecule spectrum in the database and using the unsuppressed water signal as an internal reference [27]. The following metabolites were included in the LCModel analysis: alanine, ascorbate, aspartate, β-hydroxybutyrate, creatine, γ-aminobutyrate (GABA), glutamine, glutamate, glutathione, glycine, glycerophosphorylcholine (GPC), glucose, lactate, *myo*-inositol, *N*-acetylaspartate, *N*-acetylaspartylglutamate (NAAG), phosphorylethanolamine (PE), phosphorylcholine (PCho), phosphocreatine, *scyllo*-inositol, and taurine. After LCModel analysis, metabolites with Cramér–Rao lower bounds (CRLBs) larger than 30% were disregarded as they did not fulfil reliability criteria. β-hydroxybutyrate and *scyllo*-inositol were excluded, and phosphorylcholine and glycerophosphorylcholine were analyzed as total choline (PCho + GPC). 

Flow-compensated FLASH with ECG and respiration triggering was used for cardiac MRI assessment with a resolution of 0.13 × 0.13 × 1 mm^3^ and one average. Positioning of the cardiac images was achieved with the help of three orientational scans. The first had three axial slices (TR = 50 ms, TE = 3.0 ms). This was followed by two separate scans each, with the slices (TR = 8.7 ms, TE = 3.2 ms, 24 time frames) orthogonal to each other so that the slices were positioned through the left and right ventricle in the first case and through the outflow tract of the left ventricle and the apex in the second case. Short-axis-view images of typically 8 slices (depending on heart size) with 24 time frames in each (TR = 8.7 ms, TE = 3.2 ms) were then acquired. The short-axis images were used for determination of ejection fractions using Segment v.3.0 [43].

ASL was conducted as previously described [44]. In brief, a reference scan for positioning was acquired with TE = 33 ms, TR = 3.5 s, resolution 100 × 100 mm^2^, FOV = 16 × 16 mm^2^ spectral bandwidth 54 kHz, and excitation and refocusing pulses of 2.1 and 1.7 ms, with a bandwidth of 2 kHz and a sharpness of 3. This was followed by measurement of CBF in one slice of 1.5 mm and at 233 × 234 mm^2^ resolution positioned in the mid-brain covering the cortex, hippocampus, and thalamus by means of a FAIR-EPI. This was complemented with reference measurements of the blood relaxation rate in three to four subjects per time point. The slices encompassed the MRS VOI locations. Analysis was performed as in Gottschalk [44].

### 4.3. Tail-Cuff Plethysmography

Blood pressure was measured in conscious mice using tail-cuff plethysmography (CODA, Kent Scientific, Torrington, CT, USA). After a one-week handling period, mice were acclimatized to the restrainers and the tail cuff for a training period of 7 days. Data were recorded once mice presented with stable readings over the course of one week. We recorded 30 inflation cycles; the first 15 cycles were regarded as acclimatization and the remaining 15 were used for blood pressure analysis.

### 4.4. Histology

After trans-cardiac perfusion with cold saline of mice under isoflurane anesthesia, brains were processed with the Rapid GolgiStain kit from FD NeuroTechnologies (Columbia, MD, USA), as previously described [45]. Neuronal dendrite networks and dendritic spine density of pyramidal neurons in the cortex and granule cells in the dentate gyrus of the hippocampus were imaged from 150 µm coronal Golgi–Cox-stained brain sections with an Eclipse Ti2 microscope (Nikon Instruments, Tokyo, Japan) and analyzed using the Sholl Analysis plugin (version 4.1.2) [46] in ImageJ (NIH, Bethesda, MD, USA) for dendrite branching (intersections at 5 µm distances from the soma), maximum dendrite length, and total dendritic length. Spine density was assessed in 3rd-branch-order dendritic segments from micrographs acquired at 100× magnification. For each mouse, measurements were made from at least 5 neurons in each brain area. 

### 4.5. Statistics

Results are presented as means ± SDs unless otherwise stated and were analyzed with Prism 9.4.0 (GraphPad, San Diego, CA, USA). After normality was demonstrated by Shapiro–Wilk testing or Kolmogorov-Smirnov testing, one-way ANOVA was used to test the effects of diet, or two-way ANOVA was used to test the effects of age, diet, and their interaction (ANOVA results for data in figures and tables are shown in Appendix A). Upon determination of significant effects of diet, Fisher’s least significant difference (LSD) test was used for independent comparisons between diet groups. Significant changes were accepted for *p* < 0.05. 

Partial least-squares discriminant (PLS) regression was applied to z-scores of metabolite profiles measured in the brains (Appendix A Table A2) using MATLAB 2022a (MathWorks, Natick, MA, USA). For this, each sample at 0, 7, and 14 months of treatment was assigned a dummy variable as a reference value (0 = low salt, and 1 = high salt). The variable importance in projection (VIP) was calculated for each metabolite in the cortex or the hippocampus. 

Endpoint measures, including cerebral blood flow (CBF), brain metabolite concentrations, and neuronal morphology, showed negligible differences between sexes and thus data from male and female mice were analyzed altogether. 

## Figures and Tables

**Table 1:** Body weight (g) and cage average weekly consumption of food (g/mouse/week) and water (mL/mouse/week). Number of mice is given in parenthesis. * *p* < 0.05, ** *p* < 0.01, *** *p* < 0.001 *vs*. ND; ^##^ *p* < 0.01, ^###^ *p* < 0.001 *vs*. HSD.

		Female			Male		
		ND	HSD	Rev	ND	HSD	Rev
Body weight (mean ± SD)	Baseline	24.9 ± 1.7 (9)	24.5 ± 0.9 (10)	25.8 ± 2.1 (10)	31.8 ± 2.0 (7)	32.0 ± 2.6 (6)	31.5 ± 4.3 (7)
	7 months	29.4 ± 3.5 (9)	24.4 ± 1.5 (10) ***	24.9 ± 2.0 (9) ***	32.5 ± 1.1 (6)	28.6 ± 2.3 (6) *	28.6 ± 3.5 (6) *
	14 months	34.7 ± 3.6 (7)	26.5 ± 1.7 (10) ***	35.6±4.2 (6) ^###^	33.8 ± 2.2 (6)	31.0 ± 2.6 (5)	33.6 ± 5.3 (4)
Food intake (range)	Month 1	19–24	24–28 *	25–28 *	24–29	30–44 **	32–53 ***
	Month 8	23–29	29–37 *	26–31	27–33	36–51 ***	32–42 * ^##^
	Month 9	21–27	26–38 *	21–24 ^##^	22–29	33–52 ***	26–33 ^###^
Water intake (range)	Month 1	24–42	58–74 **	50–68 **	27–39	73–106 ***	68–95 ***
	Month 8	26–43	88–118 ***	27–45 ^###^	30–35	92–120 ***	32–39 ^###^

**Table 2:** Mean arterial pressure and heart rate in beats/minute (bpm) measured by tail-cuff plethysmography (n = 10–16) and left ventricle ejection fraction, as estimated by cardiac MRI (n = 9–12). Data shown as means ± SDs. Number of mice in parenthesis.

		ND	HSD	Rev
MAP (mm Hg)	Month 6	103 ± 10 (15)	102 ± 7 (15)	99 ± 12 (16)
	Month 9	101 ± 8 (15)	102 ± 8 (14)	101 ± 8 (15)
	Month 12	99 ± 11 (14)	94 ± 8 (13)	93 ± 6 (10)
Ejection fraction (%)	Baseline	70 ± 9 (11)	72 ± 7 (12)	67 ± 6 (12)
	7 months	69 ± 7 (11)	66 ± 10 (10)	69 ± 5 (10)
	14 months	65 ± 7 (10)	69 ± 8 (9)	67 ± 6 (10)
Heart rate (bmp)	Baseline	605 ± 54 (12)	600 ± 38 (12)	614 ± 51 (12)
	7 months	600 ± 47 (11)	589 ± 50 (10)	580 ± 33 (10)
	14 months	564 ± 41 (11)	543 ± 41 (11)	548 ± 27 (11)

## Data Availability

All data are contained within the manuscript and can be shared upon request to the corresponding author.

## Abbreviations

CNS, central nervous system; ND, normal-salt diet; HSD, high-salt diet; PLS, partial least squares; Rev, reversed diet; SD, standard deviation, TCA, tricarboxylic acid.

## Appendix A

**Table A1:** ANOVA results for data in table and figures.

*Table 1*			
Body weight, females:	age F(2,71) = 61.90, *p* < 0.001;	diet F(2,71) = 27.89, *p* < 0.001;	interaction F(4,71) = 11.85, *p* < 0.001
Food intake, females:	age F(2,21) = 4.91, *p* = 0.020;	diet F(2,21) = 8.64, *p* = 0.002;	interaction F(4,21) = 2.43, *p* = 0.080
Water intake, females:	age F(1,12) = 1.22, *p* = 0.290;	diet F(2,12) = 43.40, *p* < 0.001;	interaction F(2,12) = 14.38, *p* < 0.001
Body weight, males:	age F(2,44) = 4.02, *p* = 0.030;	diet F(2,44) = 2.47, *p* = 0.100;	interaction F(4,44) = 1.20, *p* = 0.320
Food intake, males:	age F(2,36) = 1.94, *p* = 0.160;	diet F(2,36) = 30.90, *p* < 0.001;	interaction F(4,36) = 5.16, *p* = 0.002
Water intake, males:	age F(1,25) = 2.69, *p* = 0.110;	diet F(2,25) = 83.87, *p* < 0.001;	interaction F(2,25) = 32.35, *p* < 0.001
*Table 2*			
Mean arterial pressure:	age F(2,118) = 6.19, *p* = 0.003;	diet F(2,118) = 1.47, *p* = 0.2339;	interaction F(4,118) = 0.543, *p* = 0.704
Heart rate:	age F(2,91) = 15.75, *p* < 0.001;	diet F(2,91) = 1.08, *p* = 0.344;	interaction F(4,91) = 0.569, *p* = 0.686
Ejection fraction:	age F(2,86) = 1.08, *p* = 0.345;	diet F(2,86) = 0.263, *p* = 0.770;	interaction F(4,86) = 0.9637, *p* = 0.432
*Figure 1*			
Figure 1C:	age F(2,77) = 2.88, *p* = 0.062;	diet F(2,77) = 6.52, *p* = 0.002;	interaction F(4,77) = 1.22, *p* = 0.310
Figure 1D:	age F(2,77) = 60.33, *p* < 0.001;	diet F(2,77) = 10.93, *p* < 0.001;	interaction F(4,77) = 1.36, *p* = 0.256
*Figure 3*			
Figure 3A:	age F(2,104) = 11.24, *p* < 0.001;	diet F(2,104) = 1.09, *p* = 0.342;	interaction F(4,104) = 0.387, *p* = 0.818
Figure 3B:	age F(2,104) = 8.89, *p* < 0.001;	diet F(2,104) = 1.14, *p* = 0.324;	interaction F(4,104) = 1.61, *p* = 0.179
Figure 3C:	age F(2,104) = 26.79, *p* < 0.001;	diet F(2,104) = 0.13, *p* = 0.882;	interaction F(4,104) = 1.62, *p* = 0.176
Figure 3D:	age F(2,104) = 3.19, *p* = 0.045;	diet F(2,104) = 2.72, *p* = 0.071;	interaction F(4,104) = 1.70, *p* = 0.156
Figure 3E:	age F(2,104) = 2.68, *p* = 0.074;	diet F(2,104) = 7.22, *p* = 0.001;	interaction F(4,104) = 2.43, *p* = 0.052
*Figure 4*			
Figure 4A:	age F(2,106) = 8.72, *p* < 0.001;	diet F(2,106) = 2.73, *p* = 0.070;	interaction F(4,106) = 2.21, *p* = 0.073
Figure 4B:	age F(2,106) = 14.13, *p* < 0.001;	diet F(2,106) = 9.86, *p* < 0.001;	interaction F(4,106) = 2.98, *p* = 0.022
Figure 4C:	age F(2,106) = 13.59, *p* < 0.001;	diet F(2,106) = 9.08, *p* < 0.001;	interaction F(4,106) = 2.83, *p* = 0.028
Figure 4D:	age F(2,106) = 3.36, *p* = 0.038;	diet F(2,106) = 0.565, *p* = 0.570;	interaction F(4,106) = 1.68, *p* = 0.168
Figure 4E:	age F(2,106) = 0.21, *p* = 0.815;	diet F(2,106) = 2.33, *p* = 0.102;	interaction F(4,106) = 1.51, *p* = 0.206
Figure 4F:	age F(2,106) = 2.92, *p* = 0.059;	diet F(2,106) = 2.27, *p* = 0.108;	interaction F(4,106) = 1.12, *p* = 0.352
Figure 4G:	age F(2,106) = 18.43, *p* < 0.001;	diet F(2,106) = 2.26, *p* = 0.110;	interaction F(4,106) = 1.19, *p* = 0.320
Figure 4H:	age F(2,106) = 26.45, *p* < 0.001;	diet F(2,106) = 19.04, *p* < 0.001;	interaction F(4,106) = 5.03, *p* < 0.001
*Figure 5*			
Figure 5B:	F(2,13) = 0.64, *p* = 0.541		
Figure 5C:	F(2,13) = 18.60, *p* < 0.001		
Figure 5D:	end radius F(2,13) = 8.24, *p* = 0.005;	total length F(2,13) = 3.16, *p* = 0.076;	# spines F(2,13) = 6.84, *p* = 0.009
Figure 5E:	end radius F(2,13) = 12.9, *p* < 0.001;	total length F(2,13) = 15.8, *p* < 0.001;	# spines F(2,13) = 15.2, *p* < 0.001

**Table A2:** Metabolite concentrations expressed as ratios to total creatine (means ± SD).

	Baseline			7 Months			14 Months		
	ND	HSD	RD	ND	HSD	RD	ND	HSD	RD
*Cortex*									
Alanine	0.079 ± 0.042	0.068 ± 0.032	0.091 ± 0.033	0.125 ± 0.050	0.057 ± 0.036	0.117 ± 0.084	0.083 ± 0.045	0.075 ± 0.038	0.077 ± 0.043
Aspartate	0.214 ± 0.042	0.230 ± 0.061	0.237 ± 0.035	0.217 ± 0.038	0.235 ± 0.043	0.231 ± 0.039	0.220 ± 0.049	0.183 ± 0.041	0.235 ± 0.045
Creatine	0.493 ± 0.054	0.491 ± 0.052	0.501 ± 0.032	0.474 ± 0.027	0.465 ± 0.045	0.477 ± 0.020	0.495 ± 0.030	0.489 ± 0.025	0.482 ± 0.029
Phosphocreatine	0.507 ± 0.054	0.509 ± 0.052	0.499 ± 0.032	0.526 ± 0.027	0.535 ± 0.045	0.523 ± 0.020	0.505 ± 0.030	0.511 ± 0.025	0.518 ± 0.029
GABA	0.163 ± 0.016	0.158 ± 0.023	0.150 ± 0.028	0.158 ± 0.029	0.158 ± 0.018	0.145 ± 0.020	0.140 ± 0.029	0.129 ± 0.025	0.144 ± 0.013
Glutamine	0.360 ± 0.040	0.363 ± 0.036	0.371 ± 0.050	0.367 ± 0.042	0.410 ± 0.063	0.430 ± 0.061	0.401 ± 0.044	0.419 ± 0.054	0.405 ± 0.039
Glutamate	1.233 ± 0.073	1.211 ± 0.074	1.249 ± 0.121	1.246 ± 0.061	1.237 ± 0.070	1.245 ± 0.096	1.161 ± 0.091	1.159 ± 0.067	1.228 ± 0.083
Glutathione	0.162 ± 0.018	0.159 ± 0.013	0.156 ± 0.012	0.148 ± 0.013	0.154 ± 0.008	0.151 ± 0.016	0.143 ± 0.012	0.147 ± 0.013	0.139 ± 0.015
Glycine	0.119 ± 0.015	0.123 ± 0.021	0.107 ± 0.019	0.122 ± 0.019	0.098 ± 0.020	0.105 ± 0.016	0.101 ± 0.043	0.093 ± 0.028	0.109 ± 0.022
*myo*-Inositol	0.505 ± 0.031	0.488 ± 0.026	0.492 ± 0.034	0.441 ± 0.03	0.447 ± 0.026	0.442 ± 0.027	0.493 ± 0.044	0.458 ± 0.026	0.459 ± 0.026
Lactate	0.231 ± 0.143	0.239 ± 0.090	0.222 ± 0.093	0.181 ± 0.082	0.198 ± 0.090	0.194 ± 0.102	0.186 ± 0.049	0.190 ± 0.041	0.156 ± 0.065
NAA	1.111 ± 0.037	1.093 ± 0.044	1.081 ± 0.059	1.086 ± 0.049	1.063 ± 0.027	1.065 ± 0.045	1.092 ± 0.048	1.073 ± 0.042	1.116 ± 0.050
Taurine	1.343 ± 0.062	1.346 ± 0.044	1.344 ± 0.064	1.308 ± 0.057	1.290 ± 0.048	1.304 ± 0.038	1.258 ± 0.104	1.261 ± 0.083	1.258 ± 0.072
Ascorbate	0.172 ± 0.032	0.178 ± 0.023	0.182 ± 0.027	0.165 ± 0.032	0.168 ± 0.032	0.148 ± 0.026	0.152 ± 0.071	0.140 ± 0.043	0.146 ± 0.046
NAAG	0.048 ± 0.022	0.049 ± 0.025	0.046 ± 0.017	0.053 ± 0.014	0.062 ± 0.016	0.051 ± 0.014	0.054 ± 0.022	0.052 ± 0.016	0.057 ± 0.016
PE	0.250 ± 0.035	0.235 ± 0.033	0.241 ± 0.026	0.218 ± 0.044	0.197 ± 0.034	0.209 ± 0.028	0.215 ± 0.054	0.180 ± 0.027	0.212 ± 0.045
Choline	0.128 ± 0.011	0.134 ± 0.011	0.131 ± 0.013	0.145 ± 0.011	0.135 ± 0.015	0.132 ± 0.009	0.156 ± 0.015	0.146 ± 0.008	0.141 ± 0.02
*Hippocampus*									
Alanine	0.133 ± 0.038	0.138 ± 0.046	0.137 ± 0.032	0.142 ± 0.035	0.125 ± 0.035	0.162 ± 0.063	0.146 ± 0.056	0.130 ± 0.021	0.131 ± 0.047
Aspartate	0.145 ± 0.045	0.126 ± 0.053	0.137 ± 0.045	0.109 ± 0.041	0.115 ± 0.032	0.118 ± 0.047	0.125 ± 0.055	0.117 ± 0.049	0.122 ± 0.028
Creatine	0.505 ± 0.032	0.519 ± 0.027	0.528 ± 0.027	0.492 ± 0.024	0.488 ± 0.028	0.478 ± 0.024	0.516 ± 0.028	0.493 ± 0.020	0.497 ± 0.031
Phosphocreatine	0.495 ± 0.032	0.481 ± 0.027	0.472 ± 0.027	0.508 ± 0.024	0.512 ± 0.028	0.522 ± 0.024	0.484 ± 0.028	0.507 ± 0.02	0.503 ± 0.031
GABA	0.175 ± 0.038	0.175 ± 0.026	0.170 ± 0.030	0.204 ± 0.049	0.187 ± 0.034	0.185 ± 0.037	0.181 ± 0.052	0.177 ± 0.032	0.163 ± 0.044
Glutamine	0.296 ± 0.043	0.290 ± 0.026	0.311 ± 0.028	0.301 ± 0.04	0.329 ± 0.051	0.356 ± 0.059	0.327 ± 0.041	0.355 ± 0.05	0.333 ± 0.046
Glutamate	0.880 ± 0.063	0.870 ± 0.066	0.873 ± 0.033	0.905 ± 0.065	0.901 ± 0.051	0.918 ± 0.088	0.867 ± 0.067	0.879 ± 0.046	0.908 ± 0.054
Glutathione	0.150 ± 0.012	0.147 ± 0.014	0.139 ± 0.012	0.149 ± 0.018	0.150 ± 0.011	0.144 ± 0.013	0.131 ± 0.016	0.143 ± 0.011	0.139 ± 0.016
Glycine	0.127 ± 0.026	0.123 ± 0.024	0.126 ± 0.028	0.118 ± 0.021	0.105 ± 0.027	0.105 ± 0.022	0.109 ± 0.025	0.104 ± 0.019	0.132 ± 0.047
*myo*-Inositol	0.581 ± 0.028	0.573 ± 0.024	0.559 ± 0.031	0.564 ± 0.032	0.550 ± 0.03	0.553 ± 0.031	0.599 ± 0.065	0.554 ± 0.031	0.570 ± 0.036
Lactate	0.276 ± 0.065	0.250 ± 0.084	0.263 ± 0.049	0.211 ± 0.066	0.226 ± 0.055	0.236 ± 0.075	0.159 ± 0.045	0.215 ± 0.038	0.170 ± 0.092
NAA	0.780 ± 0.041	0.785 ± 0.022	0.780 ± 0.037	0.812 ± 0.032	0.804 ± 0.030	0.798 ± 0.041	0.783 ± 0.037	0.784 ± 0.031	0.783 ± 0.037
Taurine	1.284 ± 0.050	1.298 ± 0.032	1.277 ± 0.057	1.295 ± 0.068	1.297 ± 0.071	1.318 ± 0.041	1.272 ± 0.065	1.309 ± 0.068	1.292 ± 0.056
Ascorbate	0.238 ± 0.046	0.230 ± 0.043	0.244 ± 0.028	0.197 ± 0.041	0.189 ± 0.039	0.191 ± 0.041	0.204 ± 0.036	0.189 ± 0.043	0.163 ± 0.037
NAAG	0.034 ± 0.018	0.033 ± 0.013	0.029 ± 0.015	0.042 ± 0.007	0.038 ± 0.013	0.041 ± 0.011	0.042 ± 0.015	0.046 ± 0.016	0.043 ± 0.016
PE	0.186 ± 0.043	0.187 ± 0.038	0.180 ± 0.026	0.196 ± 0.026	0.179 ± 0.028	0.180 ± 0.042	0.192 ± 0.040	0.169 ± 0.028	0.208 ± 0.063
Choline	0.101 ± 0.026	0.131 ± 0.029	0.112 ± 0.023	0.142 ± 0.034	0.114 ± 0.025	0.132 ± 0.028	0.157 ± 0.039	0.133 ± 0.035	0.109 ± 0.052
